# Statistical Properties of Electron Curtain Precipitation Estimated With AeroCube‐6

**DOI:** 10.1029/2020JA028462

**Published:** 2020-12-01

**Authors:** M. Shumko, A. T. Johnson, T. P. O'Brien, D. L. Turner, A. D. Greeley, J. G. Sample, J. B. Blake, L. W. Blum, A. J. Halford

**Affiliations:** ^1^ Department of Physics Montana State University Bozeman MT USA; ^2^ NASA's Goddard Space Flight Center Greenbelt MD USA; ^3^ Space Science Applications Laboratory The Aerospace Corporation El Segundo CA USA; ^4^ Johns Hopkins Applied Physics Laboratory Laurel MD USA

## Abstract

Curtain precipitation is a recently discovered stationary, persistent, and latitudinally narrow electron precipitation phenomenon in low Earth orbit. Curtains are observed over consecutive passes of the dual AeroCube‐6 CubeSats while their in‐track lag varied from a fraction of a second to 65 s, with dosimeters that are sensitive to >35‐keV electrons. This study uses the AeroCube‐6 mission to quantify the statistical properties of 1,634 curtains observed over 3 years. We found that many curtains are narrower than 10 km in the latitudinal direction with 90% narrower than 20 km. We examined the geographic, magnetic local time, and geomagnetic dependence of curtains. We found that curtains are observed in the late‐morning and premidnight magnetic local times, with a higher occurrence rate at premidnight, and curtains are observed more often during times of enhanced Auroral Electrojet. We found a few curtains in the bounce loss cone region above the North Atlantic, whose electrons were continuously scattered for at least 6 s. Such observations suggest that continuous curtain precipitation may be a significant loss of >35‐keV electrons from the magnetosphere into the atmosphere. We hypothesize that the curtains observed in the bounce loss cone were accelerated by parallel electric fields, and we show that this mechanism is consistent with the observations.

## Key Points

- The dual AeroCube‐6 CubeSats are used to identify stationary, narrow in latitude, and persistent >35‐keV electron curtain precipitation
- Ninety percent of the observed curtains in low Earth orbit are narrower than 20 km in the latitudinal direction
- Some curtains continuously precipitated into the atmosphere for multiple seconds

## 1 Introduction

Energetic particle precipitation into Earth's atmosphere plays a fundamental role in controlling the dynamics of Earth's magnetosphere and the ionization of Earth's upper atmosphere (e.g., Millan & Thorne, 2007; Randall et al., 2015). Even though particle precipitation, and its impact on the magnetosphere and the atmosphere, has been extensively studied since the 1960s, there are precipitation phenomena that are still poorly understood. One such form of precipitation that we have limited knowledge of are electron curtains.

Electron curtain precipitation is observed in low Earth orbit (LEO) and appears stationary in latitude. Curtains were recently discovered by Blake and O'Brien (2016) using the >35‐keV electron dosimeters onboard the dual AeroCube‐6 (AC6) CubeSats that operated together between 2014 and 2017. Curtains are narrow in latitude and persist for up to at least a minute between subsequent satellite passes (the estimated curtain lifetime is ultimately limited by the AC6 separation). This discovery was made possible by AC6's actively maintained in‐track separation that varied between a few hundred meters and a few hundred kilometers. Besides the Blake and O'Brien (2016) study, not much is known about curtains including what they are, how are they generated, and their impact on the atmosphere. Answering these questions is an essential next step toward a more complete understanding of how curtains, and particle precipitation in general, affect the magnetosphere and Earth's atmosphere.

In LEO, curtains are narrower than a few tens of kilometers in the latitudinal direction. A polar‐orbiting LEO satellite, such as AC6, will pass through the curtain cross‐section in a few seconds, which appears in the electron count time series as short enhancements in flux. AC6 also observes similar‐looking transient precipitation called electron microbursts. Curtains and microbursts are observed in the AC6 data but appear short lived for different reasons: Microbursts are temporal, while curtains are spatial. Hence, AC6 and other recently developed multi‐spacecraft missions are necessary to identify and distinguish between transient microbursts and the persistent curtains.

Since the mid‐1960s, microbursts have been observed by high‐altitude balloons where they appear as sharp peaks in flux with a sub‐second duration (e.g., Anderson & Milton, 1964; Brown et al., 1965; Parks, 1967). Because balloons are relatively stationary, a microburst is easily classified as a transient phenomenon. Microburst electrons have also been directly observed by LEO satellites such as the Solar Anomalous and Magnetospheric Particle Explorer (e.g., Blake et al., 1996; Douma et al., 2017; Lorentzen, Blake, et al., 2001; O'Brien et al., 2003). But a flux enhancement that looks like a microburst from a single LEO satellite is ambiguous—it can be transient or it can be persistent, stationary, and narrow in latitude. Thus, multi‐spacecraft missions such as the Focused Investigations of Relativistic Electron Burst Intensity, Range, and Dynamics (FIREBIRD‐II) (Crew et al., 2016; Johnson et al., 2020) and AC6 (Blake & O'Brien, 2016; O'Brien et al., 2016) are necessary to resolve the temporal versus spatial ambiguity. In a case study, Anderson et al. (2017) used the Balloon Array for Radiation‐belt Relativistic Electron Losses (BARREL), together with the AC6 and FIREBIRD‐II CubeSats to resolve the spatial and temporal ambiguity of the macroscopic microburst and curtain precipitation region. Anderson et al. (2017) found that both curtains and microbursts were observed during the same radiation belt pass. While this study focuses on curtain precipitation, microburst precipitation observed across the dawn sector by AC6 was studied in Shumko et al. (2020).

While the impact of curtains on the magnetosphere and Earth's atmosphere is unknown, the impact of microbursts has been estimated to be substantial. Lorentzen, Looper, et al. (2001), Thorne et al. (2005), Breneman et al. (2017), and Douma et al. (2019), among others, estimated that microbursts could deplete the outer radiation belt electrons in about a day. Furthermore, Seppälä et al. (2018) modeled a 6‐hr microburst storm and concluded that microbursts depleted mesospheric ozone by roughly 10%. Microbursts and curtains can be easily misidentified from a single satellite; if curtains are numerous, then the atmospheric and magnetospheric impact associated with microburst observations from single satellites may be overestimated.

Precipitation bands, sometimes also referred to as spikes (e.g., Imhof et al., 1991), are a similar form of precipitation. Precipitation bands also appear as stationary and narrow flux enhancements with a >1‐s duration and can persist from an hour to as much as half a day (e.g., Blake et al., 1996; Brown & Stone, 1972). Blum et al. (2013) identified two precipitation bands and estimated that only 20 precipitation bands can deplete all of the outer radiation belt electrons. The mechanism responsible for scattering precipitation band electrons at high *L* is believed to be field line curvature scattering, but the scattering mechanism for band electrons at lower L shells is unknown. A few proposed scattering mechanisms include wave‐particle interactions and acceleration due to parallel direct current (DC) potentials (Hoffman & Evans, 1968). Precipitation bands and curtains could be related. However, a comparison with existing observations is difficult because the most recent, and the most comparable, precipitation band studies looked at relativistic precipitation bands—a very different energy regime from AC6.

The apparent curtain fine structure (shown below) that persist for at least a few seconds be explained by at least two mechanisms. In one mechanism, curtain electrons are scattered via gyroresonant interactions with chorus waves and initially produce a bounce phase structure—a bouncing packet. During subsequent bounces, the bouncing packet structure is dispersed along the bounce path and is then observed by AC6's integral channel downstream as a stable structure. Alternately, pitch angle transport occurs continuously on the local field line. The two AC6 spacecraft then pass through the same field line while the source is active. We will return to the second hypothesized mechanism later and for now focus on the first mechanism that was proposed by Blake and O'Brien (2016).

Blake and O'Brien (2016) proposed a hypothesis that curtains are drifting remnants of microbursts. If a microburst is not completely lost in the atmosphere after the initial scatter, the remaining microburst electrons will spread out (bounce phase disperse) along the entire magnetic field line over a few bounce periods. Concurrently, these electrons drift to the east, with higher‐energy electrons drifting at a faster rate. Assuming this hypothesis, the initially localized microburst is spread out in longitude into the shape of a curtain. A similar phenomenon was hypothesized by Lehtinen et al. (2000) who predicted that drifting curtains can be created by energetic runaway beams driven by lightning, but these curtains would be observed at relatively low L shells. The curtain shape and nomenclature are a direct consequence of the above untested hypotheses; however, the true shape of the precipitation studied here is unknown. To be consistent with Blake and O'Brien (2016), we also adopt the curtain nomenclature, but we stress the need to be cognizant of other possible realities and not let the curtain concept bias our interpretations.

This study expands on Blake and O'Brien (2016) by examining the statistical properties of curtains. We use 1,634 confirmed curtain observations to study the distributions of the curtain: width in latitude, the geomagnetic conditions favorable to curtains, the occurrence frequencies in latitude‐longitude, and the occurrence frequencies in *L* and MLT. Lastly, we will show examples of curtains that continuously precipitated in the bounce loss cone (BLC) region.

## 2 Instrumentation

The AC6 mission was a pair of 0.5U (10 × 10 × 5 cm) CubeSats built by The Aerospace Corporation and designed to measure the electron and proton environment in LEO (O'Brien et al., 2016, 2019). AC6 was launched on 19 June 2014 into a 620 × 700 km, 98° inclination orbit. The AC6 orbit over the 3‐year mission lifetime was roughly dawn‐dusk and precessed only a few hours in MLT: 8–12 MLT in the dawn and 20–24 MLT in the dusk sectors. The two AC6 spacecraft, designated as AC6‐A and AC6‐B, separated after launch and were in proximity for the duration of the 3‐year mission—maintained by an active attitude control system. The attitude control system allowed them to actively control the amount of atmospheric drag experienced by each AC6 unit using the surface area of their solar panel “wings.” By changing their orientation, AC6 was able to maintain a separation between 2 and 800 km, confirmed by the Global Positioning System. The two AC6 units were in a string of pearls configuration, so one unit, typically unit A, was leading the other by an in‐track lag: the time it would take the following spacecraft to catch up to the position of the leading spacecraft. To convert between the AC6 in‐track separation and in‐track lag, the AC6 orbital velocity was used. AC6's orbital velocity was 7.6 km/s and varied by as much as 0.1 km/s. The in‐track lag is readily available in the data files with the Global Positioning System, which makes it possible to remove the spatiotemporal ambiguity that affects single‐spacecraft measurements.

**Figure A1:**
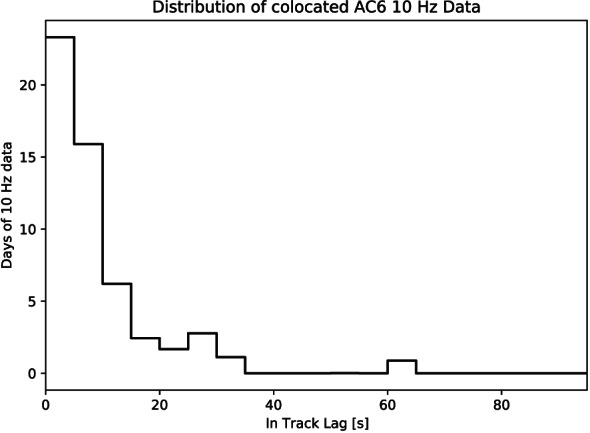
The distribution of colocated 10‐Hz data as a function of in‐track lag. Bins are 5 s wide.

Each AC6 unit contains three Aerospace microdosimeters (licensed to Teledyne Microelectronics, Inc) that measure the electron and proton dose in orbit (O'Brien et al., 2016). The dosimeter used for this study is dos1 with a >35‐keV integral electron response, as the other dosimeters either responded primarily to protons or were not identical between units A and B. All dosimeters sample at 1 Hz in survey mode and 10 Hz in burst mode; 10‐Hz data are available from both AC6 units from June 2014 to May 2017 while their in‐track lag was less than 65 s. Figure A1 shows the distribution of 10‐Hz data as a function of AC6 in‐track lag.

## 3 Methodology

### 3.1 Curtain Identification

The 10‐Hz data were used to identify curtains using the following two criteria: a high spatial correlation and a prominent peak. Before we applied the identification criteria, the AC6‐B time series was shifted by the in‐track lag to spatially align it with the AC6‐A time series.

The first identification criterion is a 1‐s rolling Pearson correlation applied to both time series. Spatial features with a correlation greater than 0.8 are considered highly correlated. The second criterion is applied to the highly correlated features to check if they are also prominently peaked. To find peaked precipitation, we used a technique similar to the technique used by Blum et al. (2015) to identify precipitation bands and by Greeley et al. (2019) to identify microbursts. Our technique quantified the number of Poisson standard deviations, *σ*, that dos1 counts in each 100‐ms bin are above a 10‐s centered running average, *b*_10_. Locations where dos1 counts are at least two *σ* above *b*_10_, in other words $\text{dos1}>2\sqrt{b_{10}}+b_{10}$, are considered prominently peaked. One bias inherent to this detection algorithm, and similar algorithms such as the burst parameter (O'Brien et al., 2003), is a reduced sensitivity for wider peaks. For a curtain with a width similar to *b*_10_, the baseline will be significantly elevated making the curtain peak less pronounced. This bias will be revisited later, but one generalization is to use a detection method that is agnostic to a wide range of peak widths, such as wavelet‐based filtering (Torrence & Compo, 1998).

**Figure 1:**
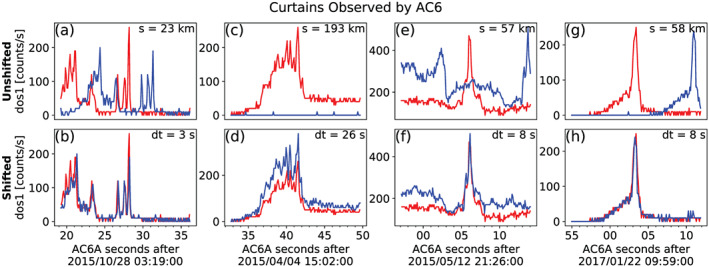
Four examples showing the >35‐keV electron time series data taken by AC6 at the same time (unshifted) in the top row and at the same position (shifted) in the bottom row, with curtains clearly visible in the bottom row. AC6‐A, whose data are shown with the red curves, was *s* kilometers ahead of AC6‐B. To show the data at the same position, the AC6‐B time series was shifted by the in‐track lag annotated by dt. These examples show that curtain precipitation was highly correlated for up to 26 s.

We tuned the automated detection parameters to identify a large number of curtains. Once curtains were automatically identified, but prior to the analysis, one author visually inspected 6,149 candidate curtains and verified 1,634 curtains. The visual inspection was performed to remove ambiguous detections where the peaks lined up in both space and time, and false positive detections that were triggered by three conditions: sharp count rate shoulders, low baseline (*b*_10_), and high correlations resulting from Poisson noise. Four curtain examples are shown in Figure 1. In each instance, the unmodified time series is shown in the top row and the spatially aligned time series is shown in the bottom row. The in‐track lag used to shift the bottom row is annotated by dt, corresponding to an AC6 in‐track separation annotated in the top row by s. The bottom row shows highly correlated curtains observed at the same location for at least 3 to 26 s. These durations are a lower bound, determined by the AC6 in‐track lag.

### 3.2 Differentiating Between Drifting and Precipitating Curtains

The AC6 dosimeters lack the necessary pitch angle resolution to differentiate between locally drifting and locally precipitating electrons, to test the Blake and O'Brien (2016) hypothesis that curtains are the drifting remnants of microbursts. Fortunately, one standard method of distinguishing between locally precipitating, drifting, and trapped particles is by using the geographic location of observations with respect to the location of the South Atlantic Anomaly (SAA).

Earth's magnetic field is asymmetric and has a region of weaker magnetic field in the South Atlantic Ocean called the SAA. The weaker magnetic field in the SAA naturally differentiates particles by pitch angle into trapped and quasi‐trapped populations. While some particles observed in LEO are trapped and will execute closed drift paths, most particles observed in LEO are quasi‐trapped: They drift around the Earth until they reach the SAA. Within the SAA, the weaker magnetic field strength can lower the particle's mirror point altitude into the atmosphere, where collisions with atmospheric neutrals and ions are more numerous and the particle is lost.

Particles that are quasi‐trapped have pitch angles in the drift loss cone and will precipitate within one drift period (often within the SAA). Particles with smaller equatorial pitch angles that are lost in the atmosphere within one bounce are in the BLC. Traditionally, a particle is in the BLC if its mirror point altitude is at or below 100 km in either hemisphere (e.g., Selesnick et al., 2003).

In most regions outside of the SAA and its conjugate point in the North Atlantic, AC6 will observe a combination of drift and BLC electrons. In the SAA, AC6 does not only observe electrons that are immediately lost, but a combination of electrons that are in the drift loss cone, BLC, and trapped (a trapped electron that locally mirrors at AC6's altitude in the SAA will mirror at higher altitudes everywhere else). In the region magnetically conjugate to the SAA in the North Atlantic, AC6 only observes electrons in the BLC. Here, if an electron makes it to AC6's altitude, it might be in the local loss cone and precipitate in the local hemisphere. Alternatively, the electron may mirror at or below AC6 and bounce to its conjugate mirror point deep in the atmosphere or below sea level in the SAA. Therefore, any electrons observed in the BLC region will likely precipitate within one bounce.

We estimated the BLC region for locally mirroring electrons in the North Atlantic Ocean using the IRBEM‐Lib magnetic field library and the Olson‐Pfitzer magnetic field model (Boscher et al., 2012; Olson & Pfitzer, 1982). We defined a latitude‐longitude grid, with a ≈0.5° × 0.5° grid size, spanning the North Atlantic at 700‐km altitude, and estimated the local magnetic field strength. The 700‐km altitude was chosen because it is the upper bound altitude for AC6's orbit and it is the conservative limit because at lower altitudes the BLC region is larger. For each latitude‐longitude point we traced the magnetic field line to the southern hemisphere and found the conjugate mirror point altitude. If the conjugate mirror point is ≤100 km, the electron is likely lost and the associated grid point is in the BLC. Furthermore, a more rigorous BLC criterion is the conjugate mirror point altitude below sea level. In this case, the electron is very likely lost. The BLC region estimated by this method closely matches the BLC region shown in Comess et al. (2013, Figure 1) and Dietrich et al. (2010, Figure 3) for other LEO satellites. Lastly, we repeated the same analysis using the Tsyganenko (1989) model, which yielded similar BLC boundaries.

## 4 Results

In this study we addressed three questions: What is the distribution of curtain widths along the AC6 orbit (mostly along the geographic latitude), when and where are curtains observed, and are curtains composed of drifting or locally precipitating electrons?

### 4.1 Curtain Width

**Figure 2:**
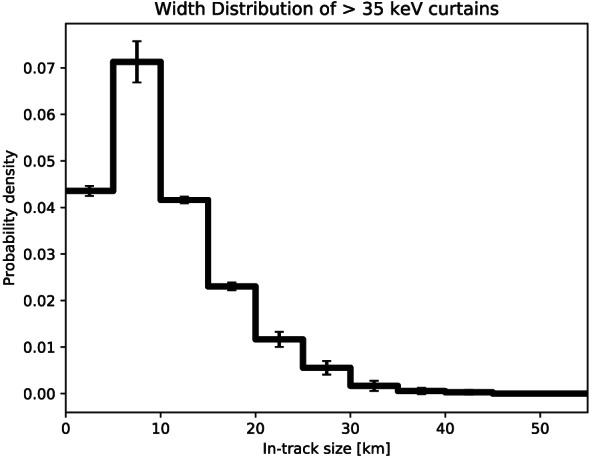
The distribution of curtain widths along the AC6 orbit. The error bars represent the Poisson standard error on the number of curtains observed in each bin.

We quantified the curtain width as the width at half of the curtain's topographic prominence, as described in Appendix B. The spatial width of a curtain is then the product of the observed width in time and AC6's orbital velocity. The curtain width is measured along AC6's orbit track which is mostly in the latitudinal direction; therefore, the estimated curtain widths are also mostly in the latitudinal direction. The distribution of curtain widths is shown in Figure 2. Curtains are very narrow. Many curtains are narrower than 10 km in the latitudinal direction, and 90% are narrower than 20 km.

### 4.2 When and Where Are Curtains Observed

**Figure 3:**
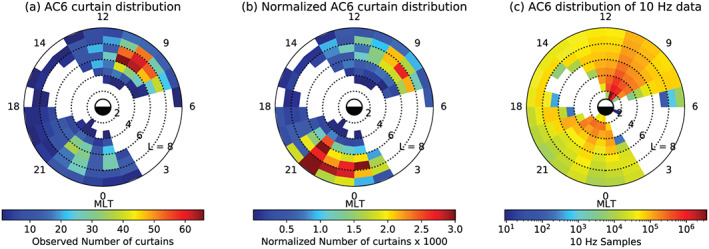
The distribution of observed curtains by L shell and MLT. Panel (a) shows the locations of all observed curtains used in this study. Panel (b) shows the curtain distribution normalized by the number of quality 10‐Hz samples taken in each bin, shown in Panel (c). The white bins in Panels (a) and (b) show where no curtains were observed, while in Panel (c) the white bins show where AC6 did not take any 10‐Hz data at the same location.

The distribution of curtains in *L* and MLT is shown in Figure 3. Figure 3a shows the distribution of the observed curtains, while Figure 3b shows the same distribution normalized by the number of quality 10‐Hz samples (flag = 0 in the data files) that both AC6 spacecraft took at the same location in each *L*‐MLT bin. In Figures 3a and 3b, the bins where no curtains were observed are white. The AC6 sampling distribution is shown in Figure 3c, whose white bins show where AC6 did not take any 10‐Hz data at the same location. The normalized curtain distribution in Figure 3b shows an enhanced curtain occurrence in the radiation belts and the plasma sheet (*L* ≈ 5–10), with the largest peak in the premidnight MLT sector. Since the AC6 dosimeters are sensitive to >35‐keV electrons, these curtains can have electron energies as low as 35 keV and thus can also be associated with the ring current or the upper energy tail of the aurora.

**Figure 4:**
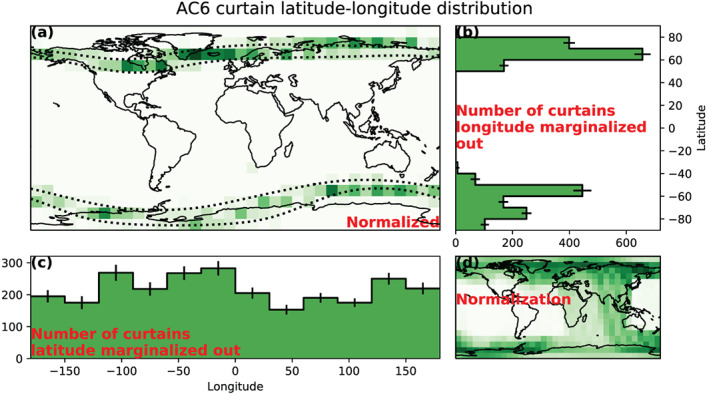
The geographic distribution of curtains. Panel (a) shows a map of the normalized number of curtains, normalized by the number of 10‐Hz samples that AC6 took a the same location, shown in Panel (d). The dotted black lines in Panel (a) show the L=4 and L=10 contours. Panels (b) and (c) show the marginalized and normalized distribution of curtains in latitude and longitude, respectively. The error bars in Panels (b) and (c) represent the standard error derived assuming Poisson statistics.

The normalized geographic distribution of curtains is shown in Figure 4a, and the marginalized distributions, summed over the marginalized variable, are shown in Figures 4b and 4c. These distributions lead us to the following three insights. First, the North‐South asymmetry seen in Figure 4b is due to the higher occurrence rate of curtains in the North Atlantic region (the BLC region). This is likely due to the typical low backgrounds in the BLC, which increases the sensitivity of our detection algorithm. Second, the number of curtains observed in the outer belt SAA in Figure 4a is low. This is in contrast to the previous point: The high trapped particle background in the SAA decreases our detection alogorithm's sensitivity. Third, Figure 4c shows that curtains are distributed roughly uniformly in longitude.

**Figure 5:**
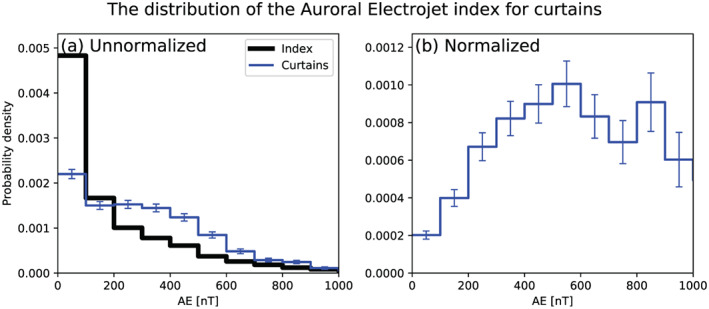
The distribution of the Auroral Electrojet (AE) index when curtains were observed. The blue line in Panel (a) shows the distribution of AE when curtains where observed, and for reference the thick black line shows the distribution of the AE index between 2014 and 2017 when quality 10‐Hz data are available from both AC6 spacecraft. Panel (b) shows the curtain distribution normalized by the AE index distribution, such that any AE index is equally probable. The error bars are derived assuming the Poisson standard error of the number of curtains observed in each AE bin.

We also examine the geomagnetic conditions favorable for curtains. Many prior wave and precipitation studies such as Douma et al. (2019) and Meredith et al. (2020) quantified the geomagnetic conditions with the Auroral Electrojet (AE) index. Therefore, we also use the AE index. Figure 5a shows the distribution of the minute cadence AE index between 2014 and 2017 in solid black, for times when quality 10‐Hz data were available from both AC6 units. The distribution of the AE index when curtains were observed is shown by the solid blue lines. Curtains are observed during both low and high geomagnetic activity, slightly more often at higher AE than the index itself (curtain distribution trends above the AE index when *AE* > 200). Lastly, we normalized the curtain distribution in Figure 5a assuming any AE index is equally probable. The normalized curtain distribution is shown in Figure 5b, which highlights the increasing curtain occurrence frequency with increasing AE, up to ≈600 nT.

### 4.3 Local Atmospheric Precipitation

**Figure 6:**
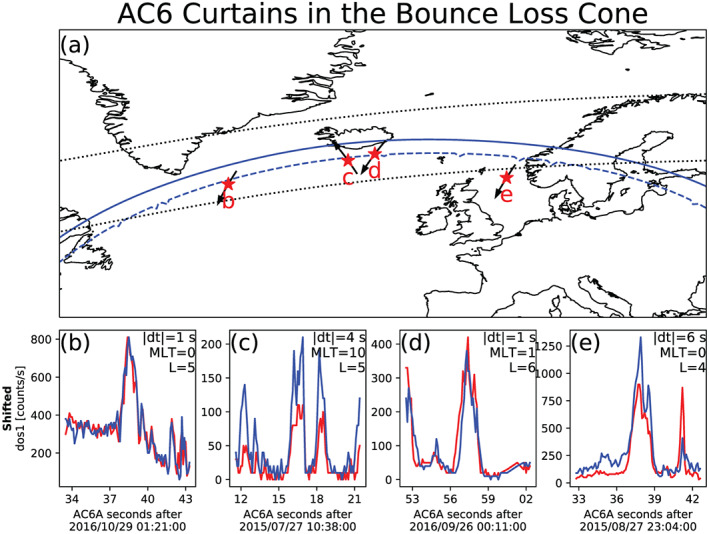
Curtains observed in the bounce loss cone region. Panel (a) shows a map of the North Atlantic region with the outer radiation belt, defined by L=4–8, shown with the dotted black curves. The solid blue curve shows the northern boundary of the bounce loss cone region. Along this curve, electrons locally mirroring at 700‐km altitude have a conjugate mirror point at 100‐km altitude in the SAA. A more strict bounce loss cone criterion is the dashed blue curve that represents a conjugate mirror point altitude at sea level in the SAA. The four red stars with labels show the locations of the curtains shown in the corresponding Panels (b)–(e). In Panel (a), the black curves with arrows that pass through the red stars show 1 min of AC6's orbit path and direction. Panels (b)–(e) show the four curtain examples with the AC6‐A data shown by the red line and the time‐shifted AC6‐B data with the blue line. The annotations in each example show the AC6 in‐track lag (dt), *L*, and MLT rounded to the nearest integer. AC6‐A was leading in all examples except in Panel (d).

Lastly, we investigate if curtains are drifting or locally precipitating. Figure 6a shows a map of the northern BLC region in the North Atlantic. The solid blue line is the northern boundary where an electron that mirrors locally at 700 km has a conjugate mirror point at 100 km in the SAA. Immediately south of the solid blue line, the conjugate mirror altitude rapidly decreases toward, and below, sea level. The dashed blue line is the boundary where the conjugate mirror point altitude is at sea level. South of this line the conjugate mirror point is inside the Earth. For reference, AC6 takes about 30 s to move between the solid and dashed blue curves. The two dotted black curves in Figure 6a are roughly the boundary of the outer radiation belt, defined as L=4–8.

Of the 1,634 curtains, we found 36 curtains that were observed inside the BLC region. Figures 6b–6e show four curtain examples (AC6‐B time shifted by the in‐track lag), along with the AC6 in‐track lag, *L* and MLT during the observations annotated. The AC6 locations where these curtains were observed are shown in Figure 6a with the red stars and the corresponding panel labels. Recall from section 3.2 that in this region, all particles that can access AC6, regardless of their pitch angle, were in the BLC. Therefore, these electrons locally precipitated within a half bounce period. The mere existence of these curtains suggests that the curtain source was actively precipitating electrons on the local field line for at least the duration of the AC6 pass through the region.

## 5 Discussion

### 5.1 Curtain Width

Curtains are narrow in latitude. Figure 2 shows that the width of most curtains is on the order of 1–3 s in time as observed by AC6, corresponding to a 8‐ to 20‐km spatial width along the AC6 orbit track. The reduced sensitivity of the detection algorithm, as described in section 3.1, is unlikely to significantly underestimate the curtain width distribution because most curtains had a width less than half of the 10‐s baseline's width. Scaled to the magnetic equator, the curtain widths correspond to a source with a radial scale size of a few hundred kilometers. The curtains with a <1‐s duration suggest that past microburst observations could have been mistaken for curtains, so the microburst impact on the atmosphere and the radiation belt is overestimated.

As shown in Figure 1, it is remarkable that some curtains maintain a fine structure after multiple seconds with little observable difference. However, sometimes curtains appear to be slightly and systematically shifted in latitude, while maintaining their fine structure (not shown).

### 5.2 When and Where Are Curtains Observed

Figure 3b shows that curtains originate on L shells corresponding to the outer radiation belt or the plasma sheet and are observed relatively more in the premidnight than late‐morning MLT regions. The MLT distribution is biased by AC6's orbit, and curtains are very likely to exist inside the sizable sampling gaps shown in Figure 3c. Furthermore, curtains are more often observed at higher L shells near midnight MLT; however, the sampling statistics at high *L* are limited because AC6 rapidly crosses high L shells.

The curtain distribution in longitude, shown in Figure 4c, does not show a clear trend. If curtains were drifting, they would precipitate into the SAA, so very few curtains will be observed just to the east of the SAA. Further to the east, the number of curtains will increase to a maximum at the western edge of the SAA. Thus, the lack of a clear longitudinal trend shows that curtains are unlikely a purely drifting phenomenon.

Lastly, Figure 5b shows that curtains are associated with an enhanced AE up to around 600 nT. While it is not a direct comparison (due to the electron energy channels and the binning scheme), Douma et al. (2019) showed that the number of microburst observed by SAMPEX also increases with increasing AE, up to about AE = 300 nT.

### 5.3 Curtains Observed in the BLC

The handful of curtains observed in the BLC, and shown in Figure 6, did not drift because in order for them to drift, they must also bounce. However, as mentioned in section 3.2, the curtain electrons observed in the BLC had a conjugate mirror point at or near the sea level so they could not bounce. Therefore, these curtains cannot be explained by the drifting microburst hypothesis presented by Blake and O'Brien (2016). One possible explanation is that the curtain electrons were observed at the end of their drift orbit. However, these curtains were seen far from the western edge of the BLC, so any drifting electrons would have been lost before AC6 observed them. The curtain precipitation persisted for multiple bounce periods (≈1.5 s for 35‐keV electrons in this region), suggesting that the curtain generation mechanism must be capable of continuously scattering electrons. Thus, we will now consider the second hypothesized mechanism introduced in section 1: a persistent source region.

### 5.4 An Alternative Curtain Generation Mechanism

One candidate mechanism that can generate curtains is a DC electric field that is parallel to the background magnetic field that lowers the electron mirror point to AC6 altitudes. To find the minimum potential, we assume the electron is barely trapped and has a 100‐km conjugate mirror point altitude in the SAA; hence, initially, the electron will mirror above AC6 in the BLC region.

To find the parallel potential, *q*Φ, we assume $W_{i}=35$‐keV electron kinetic energy at its initial mirror point with a magnetic field strength of *B*_*i*_. The kinetic energy at the initial mirror point can be written as $W_{i}=\mu B_{i}$ where *μ* is the first adiabatic invariant that is conserved during this acceleration. When a parallel potential accelerates the electron of charge *q* and does *q*Φ amount of work, the electron will mirror closer to Earth's surface, at a field strength *B*_*f*_. The electron's final energy is then $W_{f}=\mu B_{f}$. Now we relate the initial and final kinetic energy of the electron:

(1) $$\mu B_{f}=\mu B_{i}+q\Phi .$$

Then we solve for *q*Φ and substitute *μ*, to express the above equation as a function of the initial kinetic energy:

(2) $$q\Phi =W_{i}\frac{\left(B_{f}-B_{i}\right)}{B_{i}}.$$

The parallel potential is proportional to *W*_*i*_, so a larger potential is necessary to accelerate higher‐energy electrons. AC6's dos1 electron energy response increases rapidly from 35 keV to a peak at 100 keV (Figure 2 in O'Brien et al., 2019); therefore, our assumption that $W_{i}=35$ keV can underestimate the parallel potential. Nevertheless, the counts observed by AC6 are a convolution of, among other things, the AC6 dos1 electron energy response and the exponentially falling electron energy spectrum. Thus, the majority of electrons that AC6 observed have energies close to 35 keV, and therefore, the $W_{i}=35$ keV is appropriate.

We again used the Olson‐Pfitzer magnetic field model to estimate *q*Φ. For each example curtain in Figure 6, we first estimated the local magnetic field, *B*_*f*_, that the electron descended to after the acceleration. Then we traced the local field line into the SAA. We estimated *B*_*i*_ at 100‐km altitude in the SAA for barely trapped electrons. With the initial and final *B*, along with $W_{i}=35$ keV, the minimum potential was between *q*Φ = 1–4 kV for the examples shown in Figure 6.

The range of estimated potentials is typical for the inverted‐V discrete aurora. Partamies et al. (2008), using observations made by the Fast Auroral SnapshoT (FAST) mission, reported that auroral inverted‐V electron precipitation structures, with electron energies up to a few tens of kiloelectron volts, were accelerated by 2‐ to 4‐kV parallel potentials. The inverted‐V structure and curtains share several similarities including latitudinal width, high occurrence rate in the midnight MLT region, and the maximum inverted‐V energy extends into tens of kiloelectron volts (e.g., Marklund et al., 2011; Partamies et al., 2008; Thieman & Hoffman, 1985). AC6's dos1, with its 35‐keV electron threshold, may be observing the narrow, highest energy tip of the inverted‐V aurora. A possible connection between the inverted‐V structures is intriguing, but by itself AC6 cannot easily test this hypothesis. To investigate further, a follow‐on study could look at ground‐based auroral imager data and look for mesoscale auroral arcs when AC6 observed curtains overhead.

Regardless of the source of the curtain precipitation, the impact of curtains on the atmosphere needs to be quantified. Even if the curtains observed in the BLC are the exception and other curtains are drifting, the drifting curtains will still precipitate within one drift period. Precipitating electrons produce odd reactive nitrogen (NO_X_) molecules that are currently underestimated by atmospheric models such as the widely used Whole Atmosphere Community Climate Model (WACCM) (e.g., Randall et al., 2015). Curtain precipitation could be one of the underestimated sources of mesospheric N*O*_*X*_. An AC6‐like mission with pitch angle and energy resolution will be necessary to quantify the curtain impact on the atmosphere.

## 6 Conclusions

The 1,634 curtains examined here allowed us to make the following inferences:

1. Curtains are narrow—90% are less than 20 km wide in the latitudinal direction.
2. Considering AC6's sampling bias to the premidnight and prenoon MLT regions, curtains occur relatively more often in the premidnight than the prenoon MLT region.
3. Curtains occur relatively more frequently during active geomagnetic periods.
4. Some curtains continuously precipitate into the atmosphere for at least multiple seconds, a lower bound duration that is set by AC6's in‐track lag.

As shown in Figure 1, curtain precipitation is narrow with a fine structure that persists for multiple seconds—for at least 26 s as shown in Figure 1d. Either the scattering mechanism that continuously generates curtains is physically static for multiple seconds, or the curtain electron drift is often undisturbed.

Evidence for the curtain‐microburst relationship, hypothesized in Blake and O'Brien (2016), is not clear. Curtains observed in the BLC cast doubt on the curtain‐microburst hypothesis. Some curtains continuously precipitate for at least a few seconds and can be a significant source of energetic electron precipitation into the atmosphere. Lastly, we found that the continuous scattering of curtain electrons can be explained by a hypothesized parallel DC electric field, possibly relating curtains to the aurora.

## Figures and Tables

**Figure B1:**
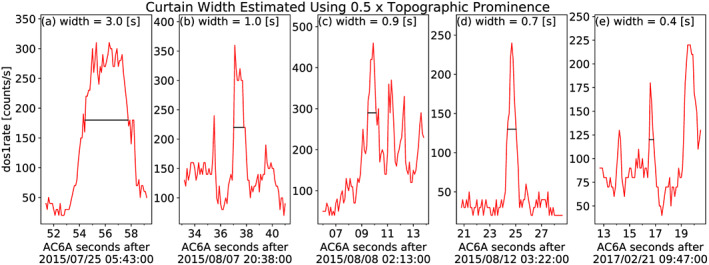
Five examples of curtains observed by AC6‐A shown with the red curves and the curtain widths are shown with the horizontal black lines and annotated. The height of the horizontal black lines is at half of the curtain's topographic prominence.

## Data Availability

The AC6 data and documentation are available at http://rbspgway.jhuapl.edu/ac6, and the IRBEM‐Lib version used for this analysis is archived online (http://doi.org/10.5281/zenodo.4075396). The 1‐min cadence provisional AE index data were downloaded online (http://wdc.kugi.kyoto‐u.ac.jp/aeasy/).

## Appendix A Distribution of Colocated 10‐Hz Data

### A.1

Figure A1 shows the distribution of colocated AC6 10‐Hz data as a function of in‐track lag. This distribution is heavily dominated by small in‐track lags, and 72% of the colocated 10‐Hz data were taken when AC6 was separated in‐track by less than 10 s, corresponding to 75‐km in‐track separation. Therefore, most of the curtains studied here were observed for small in‐track lags, which limits our ability to explore the extent of the curtain duration.

## Appendix B Estimating Curtain Widths

### B.1

The curtain width in the dos1 time series is defined here as the width at half of the curtain's topographic prominence. Topographic prominence for a peak in a time series is the height of a peak relative to the maximum of the two minima on either side of the peak. The minima on either side of the peak are searched for between the peak and the nearest higher peak on that side. Figure B1 shows five examples of curtains observed by AC6‐A in red (for clarity the AC6‐B data are not shown), and the curtain width is shown by the horizontal black line.
